# Social Dysfunction and Neural Processing of Emotional Valence Across Depressive and Anxiety Disorders

**DOI:** 10.1155/2024/8564344

**Published:** 2024-10-10

**Authors:** Simon Braak, Geor Bakker, Tanja Su, Channah Osinga, Laura Nawijn, Marie-Jose van Tol, Nic J. A. Van der Wee, Yolande Pijnenburg, Brenda W. J. H. Penninx

**Affiliations:** ^1^Department of Psychiatry, Amsterdam UMC, Location Vrije Universiteit Amsterdam, Boelelaan 1117, Amsterdam, Netherlands; ^2^Amsterdam Neuroscience, Mood, Anxiety, Psychosis, Sleep and Stress and Neurodegeneration Programs, Amsterdam, Netherlands; ^3^Amacrine Scientific Consulting, Amsterdam, Netherlands; ^4^Department of Clinical Psychology, Leiden University, Wassenaarseweg 52, Leiden, Netherlands; ^5^Department of Psychiatry, Amsterdam UMC, Location University of Amsterdam, Meibergdreef 9, Amsterdam, Netherlands; ^6^Cognitive Neuroscience Center, University Medical Center Groningen, University of Groningen, Groningen, Netherlands; ^7^Department of Psychiatry, Leiden University Medical Centre, Leiden, Netherlands; ^8^Alzheimer Center Amsterdam, Neurology, Vrije Universiteit Amsterdam, Amsterdam UMC location VUmc, Amsterdam, Netherlands

**Keywords:** anxiety, depression, facial emotional processing, negative valence, positive valence, social dysfunction

## Abstract

Social dysfunction is common across psychiatric disorders, including depressive and anxiety disorders. Both disorders have been associated with negative biases in socioaffective neural processing, which may impact responses to social stimuli. This study aims to determine whether social dysfunction across these psychiatric disorders is indeed coupled to altered neural processing of negative and positive valenced emotional stimuli and whether a common neurobiological correlate can be identified. An implicit emotional faces functional magnetic resonance imaging task was used to measure brain activation in response to emotional stimuli in participants with depression (*N* = 46), anxiety (*N* = 45), comorbid depressive and anxiety disorders (*N* = 57), and healthy controls (*N* = 52). Social dysfunction was indexed using five items of the World Health Organisation Disability Assessment Schedule-2.0 (i.e., perceived social disability) and with the De Jong-Gierveld Loneliness scale (LON; i.e., perceived loneliness). Higher perceived social disability scores were associated with greater brain activation in the left angular gyrus in response to sad emotional faces across all participants but did not correlate with responses to overall negative (sad, angry, and fearful) or positive (happy) emotional faces. No interaction effect of diagnosis was observed for the finding. Perceived loneliness scores did not correlate with brain activation to emotional faces. Taken together, perceived social disability across persons with and without depressive and/or anxiety disorders converges specifically on sad emotional processing of the left angular gyrus, suggesting a potential common neurobiological correlate for social dysfunction.

## 1. Introduction

Current diagnostic classifications of psychiatric disorders, including depressive and anxiety disorders, rely on assessments of clinical presentation [1]. These psychiatric classifications are, however, not underpinned by neurobiological systems and do not take into account the heterogeneity within and across these psychiatric disorders [1, 2]. As a result, research findings are often inconsistent, and treatment effects are insufficient [3]. Consequently, alternative frameworks have emerged to study psychiatric disorders, such as the Research Domain Criteria (RDoC) framework [2]. The RDoC framework aims to understand mental disorders based upon varying degrees of dysfunction in (neuro)biological and psychological domains that cut across diagnostic boundaries (i.e., transdiagnostic) [2]. In this way, the entire spectrum of functioning is studied; from normal range to increasingly more dysfunction [4]. Previous research has often used the term “transdiagnostic” to indicate associations that can be found across two or more disease classes and healthy controls [5–8]. This approach encourages a shift toward a biologically-based perspective and aims to advance precision medicine approaches that target underlying pathophysiology [2, 9].

An important transdiagnostic domain suggested within the RDoC framework is the social processes domain and includes, but is not limited to, affiliation and attachment and the perception and understanding of others [10]. Social dysfunction is one of the first and most common feature of many (neuro)psychiatric disorders, such as depressive and anxiety disorders, and greatly affects the patient's quality of life and mortality risk [5, 11–14]. In both disorders, affective aspects of social functioning (e.g., perceived social disability and feelings of loneliness) might be more impaired than behavioral ones (e.g., network size and social activities) [11, 15]. Symptom severity, including social dysfunction, is often worse in comorbid depressive and anxiety (comorbid) patients compared to patients with single depressive or anxiety disorders [11, 16–18]. Even in remitted patients, aspects of social functioning can remain impaired [11, 19, 20]. Importantly, higher perceived social disability is a predictor for poor functional outcomes 2 years later in depressive and anxiety disorders [11]. The high degree of overlap in social dysfunction across depressive and anxiety disorders may indicate shared underlying neurobiological mechanisms [11].

The RDoC approach has focussed on various brain regions that process positive stimuli and those that process negative stimuli, known as the positive and negative valence systems [21]. Activation of the positive valence system involves regions associated with responses to positive motivational contexts and dopamine release, such as the nucleus accumbens, orbitofrontal cortex (OFC), and ventromedial prefrontal cortex [21–24]. Activation of the negative valence system, however, often involves limbic areas, such as the amygdala, and the insular cortex [21, 24, 25]. The implicit emotional faces fMRI task can be used to investigate how these brain regions respond to positive and negative socioemotional stimuli, contributing to emotional experiences, motivational states, and adaptive behaviors linked to social functioning [26]. For instance, blunted activation of the positive valence system to pleasant stimuli in depressive disorders has been linked to anhedonia [27]. Heightened activation in the negative valence system, particularly in the amygdala, has also been commonly observed in depressive and anxiety disorders [28]. The heightened sensitivity to negative stimuli and diminished sensitivity to positive stimuli, indexed by positive/negative valence temperament, have been associated with more perceived social disconnectedness across depressive and anxiety disorders [29].

Our previous study showed that behavioral aspects of social dysfunction (such as social withdrawal and interpersonal functioning), but not perceived loneliness, were coupled to greater activation in fronto–parieto–limbic brain regions in response to sad emotional faces across schizophrenia, Alzheimer's disease, and healthy controls, along with reduced activation in those brain regions in response to happy emotional faces [30]. These fronto–parieto–limbic brain regions encompass areas associated with social pain (e.g., social rejection) and mentalizing, such as the amygdala, insula, anterior cingulate cortex (ACC), and inferior parietal lobe (IPL) [5, 30]. Interestingly, social dysfunction did not correlate with any brain activation in response to fearful emotional faces, indicating that persons experiencing social dysfunction might have a negative emotional bias that is more specific to sad emotions [30]. However, it remains unclear whether there is a similar common factor within positive and negative valence systems that correlates with social dysfunction across depressive and anxiety disorders. These data are important to explore the effectiveness of therapeutic strategies targeting specific brain regions to improve social dysfunction. Therefore, in the current study, we explored whether a common neurobiological correlate for social dysfunction could be found in the neural circuitry underlying negative and positive valence processing across patients with depressive, anxiety, comorbid disorders, and healthy controls. Based on prior work, we hypothesized that greater social dysfunction would be associated with greater activation in fronto–parieto–limbic brain regions in response to sad emotional valence, along with reduced activation in these brain regions in response to positive valence, irrespective of diagnosis [30].

## 2. Methods and Materials

### 2.1. Participants

All data were acquired in the Netherlands Study of Depression and Anxiety (NESDA) study, which is an ongoing longitudinal cohort study that investigates the course and etiology of depressive and anxiety disorders [31]. Between 2005 and 2007, 301 participants from the NESDA main sample (*N* = 2981), aged between 18 and 57 years, took part in the neuroimaging ancillary study [32]. This study was conducted at the university medical centers in Amsterdam (AMC), Leiden (LUMC), and Groningen (UMCG). NESDA was approved by the Medical Ethical Review Boards of all participating centers and all participants provided written informed consent. All study procedures were performed in accordance with the principles of the Declaration of Helsinki.

The DSM-IV Composite International Diagnostic Interview (Version 2.1 [33]) was performed by a trained clinician to confirm the presence of a depressive (major depressive disorder or dysthymia) and/or an anxiety disorder (panic disorder, social phobia, generalized anxiety disorder, or agoraphobia) in the past 6 months in patients, or absence of a lifetime DSM-IV diagnosis in healthy controls. In addition, the severity of depressive symptoms over the past week was assessed by the Inventory of Depressive Symptoms (IDS)-self-report questionnaire, while severity of anxiety symptomatology was assessed by the Beck Anxiety Index (BAI) [34, 35].

Exclusion criteria for NESDA included an insufficient command of the Dutch language to undergo the assessments and the presence of other clinically diagnosed primary psychiatric disorders (e.g., obsessive–compulsive disorder, psychotic disorder, bipolar disorder, or severe addictive disorders). Additional exclusion criteria for the neuroimaging study were (1) history of seizures and brain injury; (2) current alcohol or substance abuse; (3) use of psychotropic medication other than stable use of selective serotonin reuptake inhibitors or serotonin-norepinephrine reuptake inhibitors and/or infrequent benzodiazepines use (with a maximum of three times a week and not within 48 h before scanning); and (4) MRI contraindications.

**Figure 1:**
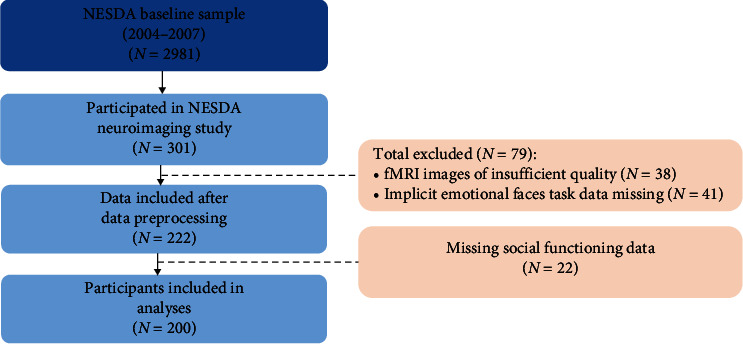
Flowchart detailing the data selection procedure.

Out of the 301 participants in the neuroimaging study, implicit emotional faces task imaging data was available for 260 participants. Participants were excluded due to insufficient image quality (*N* = 38) (e.g., due to movement (> |2.5| mm/|0.4|rad)) or missing social functioning data (*N* = 22), resulting in a final sample of 200 participants (*N* = 46 depressive disorder; *N* = 45 anxiety disorder; *N* = 57 comorbid; and *N* = 52 healthy controls) (Figure 1).

### 2.2. Social Dysfunction

Perceived social disability was measured using four items from the “getting along” subscale and one item from the “participation” subscale from the self-report World Health Organization Disability Assessment Schedule-2.0 (WHODAS), similar to previous studies [36–39]. The WHODAS scale has been developed to capture the level of functioning and disability in six major life domains [36]. It is a valid and reliable tool to measure disability across different populations [36]. The “getting along” subscale captures difficulties with interacting with strangers, maintaining friendships, getting along with people you know well, and making new friends. The “participation” subscale includes a question about the extent to which a person has difficulty with participating in community activities. All items of the WHODAS reflect functioning over the preceding 30 days [36]. The items were scored on a five-point Likert scale, ranging from 0 (“no difficulty”) to 5 (“significant difficulties/being unable”), resulting in a sum score (range 5–25) with higher scores indicating higher perceived social disability.

Subjective feelings of loneliness were measured using the De Jong-Gierveld Loneliness (LON) self-report questionnaire, which is shown to be a valid and reliable instrument [40, 41]. The questionnaire consists of 11 statements, such as “I miss the pleasure of the company of others,” that have to be answered on three-point Likert scales (1 = yes, 2 = more or less, 3 = no). Following the scoring guidelines, the answers were dichotomized to reflect the presence or absence of loneliness symptoms. This resulted in a total sum score (range 0–11), with higher total scores indicative of higher perceived loneliness.

The WHODAS (e.g., the five items to assess perceived social disability) and LON scale were moderately correlated with each other (Spearman's *r* = 0.64, *p* < .0001) suggesting that while having some overlap they capture partly different aspects of social dysfunction.

### 2.3. Implicit Emotional Faces Task

**Figure 2:**
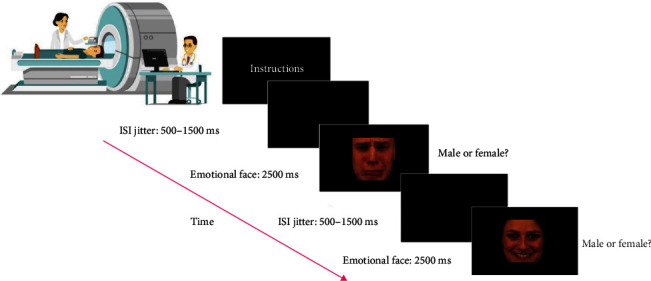
Time course of stimulus presentation for the implicit emotional faces task during the scanning session. ISI, interstimulus interval. Figure adapted from Braak et al. [30].

The implicit emotional faces paradigm (Figure 2) has been described before by Demenescu et al. [42]. Color photographs of sad, fearful, angry, happy, and neutral facial expressions were presented to participants together with scrambled faces. Photographs were selected from the Karolinska Directed Emotional Faces System [43]. Twenty-four photographs were selected for each of the five facial expressions, comprising 12 female and 12 male faces, and 80 scrambled faces. Photographs were presented in a pseudorandom order, ensuring that no more than two faces were shown before a scrambled face was presented, with a total of 200 stimuli. Each photograph was presented on the screen for 2.5 s with a black screen interval varying between 0.5 and 1.5 s, bringing the total task duration to ~7 min and 40 s. Participants were asked to indicate the gender of the depicted faces through a button press to ensure task engagement. During the presentation of scrambled faces, participants had to press left or right buttons in accordance with the on-screen instructions.

### 2.4. MRI Data Acquisition and Preprocessing

Imaging data were acquired on Philips 3T MRI scanners equipped with a SENSE-8 (LUMC and UMCG) or SENSE-6 (AMC) channel head coil. Imaging parameters and preprocessing methods are described in detail elsewhere [44]. T1-weighted anatomical MRI scans were acquired with the following parameters: repetition time (TR) = 9 ms, echo time (TE) = 3.5 ms, flip angle = 8°, field of view (FOV) matrix size = 256 × 256, and voxel size = 1 × 1 × 1 mm. Subsequently, T2⁣^*∗*^-weighted gradient-echo echoplanar imaging scans were acquired for the emotional faces task (AMC and LUMC: 200 whole brain volumes, TR = 2300 ms, TE = 30 ms, flip angle = 80°, 35 axial slices, no slice gap, FOV = 220 × 220 mm; voxel size = 2.3 ×2.3 mm, slice thickness = 3 mm); UMCG similar except: TE = 28 ms, 39 axial slices, voxel size = 3.45 × 3.45 mm. The imaging data were preprocessed using FSL 5.0.8 and included artifact removal using FSL FIX [45], motion correction (realignment), grand mean scaling, spatial smoothing with 6 mm Gaussian kernel, motion artifact removal using independent component analysis-based automated removal of motion artifacts (ICA-AROMAs [46]), and high-pass filtering (cut-off 80 s). The first-level statistical maps were registered to Montreal Neurological Institute (MNI) standard space using boundary-based registration with the registration matrices obtained from the first coregistration of the functional image to T1 and registration of T1 images to MNI space. Participants were excluded if movement was > |2.5 | mm/|0.4| rad, or if imaging quality was insufficient. Two researchers conducted quality control and participants were excluded if both researchers assessed the imaging quality as insufficient.

### 2.5. Data Analyses

Group differences in demographic and clinical data were compared in R (Version 4.2.1) using *χ*^2^ for dichotomous variables and analysis of variance for continuous variables. The independent Kruskal–Wallis test was used as a nonparametric test when assumptions for parametric testing, that is, normality and/or homogeneity of variance, were not met. When a significant difference was found, post hoc pairwise comparisons were executed to determine which groups differed.

Subject-level statistical analyses were performed using a general linear model (GLM) with delta functions to model responses to negative and positive valence. The following contrasts were tested to measure the neural responses to the emotional faces in these subject-level GLMs: angry vs. scrambled faces [1, −1], sad vs. scrambled faces [1, −1], fear vs. scrambled faces [1, −1], and happy vs. scrambled faces [1, −1]. Scrambled faces rather than neutral faces were used based on previous literature showing that patients with depression and anxiety can misattribute neutral faces as having negative valence [47–49]. Using scrambled faces was considered to minimize the risk of underestimating true negative valence responses or confounding positive ones. This decision does imply that the results presented may include some nonemotional variance. First, a GLM analysis for the sad emotional stimuli was conducted, as we previously demonstrated a notable association between social dysfunction and the processing of sad emotional stimuli in fronto–parieto–limbic brain regions across schizophrenia, Alzheimer's disease, and healthy controls [30]. The RDoC domain of negative valence systems includes constructs of negative affect that are strongly interrelated such as threat (fear), loss (sadness), and frustrative nonreward (anger) [50]. Therefore, we subsequently tested whether the potential relationship between social dysfunction and brain activation in response to sad emotional faces is due to an overall negative valence bias, including angry and fearful emotional faces. This was tested by a mixed model using FLAME 1 + 2 treating subjects as random factor and emotional stimulus type, fear, sad, and anger as fixed effect. Finally, a separate model was used to test associations between positive valence system activation elicited by the happy emotional faces and social dysfunction scores treating subject as random effect. All GLMs included the individual participant's WHODAS or LON score as a regressor, wherein separate contrasts (contrasts of parameter estimates) were defined to probe the effects of these two constructs on task-related activity across the sample. This model included diagnostic nosology as a categorical variable (depressive, anxiety, comorbid disorders, and healthy controls) and demographic factors (age, sex, years of education, and scan site) as covariates.

Post hoc analyses that assessed diagnosis × WHODAS or LON interaction effects or medication effects were performed in case WHODAS or LON scores were significantly associated with brain activity in response to negative or positive valence. Here, we first extracted the mean neural activation of the significant clusters and subsequently examined whether the slopes of the association between neural activity and social dysfunction scores were similar across the four groups (depressive, anxiety, comorbid disorders, and healthy controls) or were confounded by antidepressant and/or benzodiazepine use. Cohen's *F*^2^ effect sizes were calculated in case WHODAS or LON scores were significantly associated with the neural activity in response to negative or positive valence [51]. For sensitivity purposes regarding negative and positive valence system activation, it was examined whether the task elicited expected brain activation in response to overall negative and positive valence and whether this activation differed between groups. This latter was done by extracting the mean neural activation of the significant clusters in response to overall negative and positive valence, and the clusters in the bilateral amygdala (in case of negative valence) and right OFC (in case of positive valence) for all participants. Analyses were performed with all independent variables demeaned across groups, with statistical thresholding and correction for multiple comparisons achieved through cluster-based thresholding (*z* = 3.1) with family-wise error rate cluster *p* threshold of 0.05. All higher level analyses (group-level) were conducted using FEAT (FSL 6.0.4) [52, 53].

## 3. Results

### 3.1. Sample Characteristics

The sample characteristics are listed in Table 1. The distribution of age and sex was similar across the four groups. The number of years of education was higher in healthy controls compared to all patient groups (*p*'s < 0.05). The number of depressive and anxiety symptoms was higher in all patient groups compared to healthy controls (*p*'s < 0.0001). Disease severity (indexed by the IDS and BAI) in the patient groups was mild to moderate. The number of depressive symptoms was higher in the comorbid group compared to the depressive and anxiety group (*p*'s < 0.05). The number of anxiety symptoms was higher in the comorbid group compared to the depressive group (*p* < 0.01) but did not differ from the anxiety group. All patient groups had higher WHODAS and LON scores compared to healthy controls (*p*'s < 0.0001), with the comorbid group scoring higher on the WHODAS and LON than the anxiety group (*p*'s < 0.01). WHODAS and LON scores did not differ between the depressive and anxiety group.

### 3.2. Relationship Between Negative and Positive Valence Processing and Social Dysfunction

**Figure 3:**
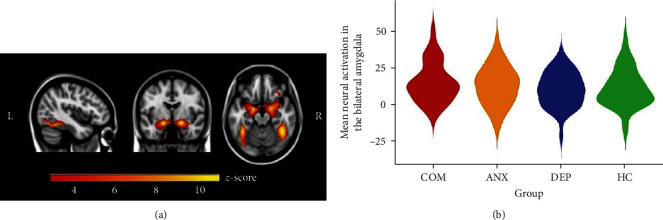
Negative valence system activation. (a) The following contrast was used for task activation: overall negative emotional valence (angry + sad + fearful emotional faces) > scrambled faces. Significant task activation in the bilateral amygdala, hippocampus, fusiform cortex, right frontal gyrus, and right orbitofrontal cortex. Threshold *z* = 3.1 for activation clusters (*x* = −42, *y* = −4, *z* = −18), overlaid on Montreal Neurological Institute 152 2 mm template. (b) The violin plot shows the distribution of the mean neural activation of the significant cluster within the bilateral amygdala in response to overall negative affect stimuli for each group. COM, comorbid depressive and anxiety disorder; ANX, anxiety disorder; DEP, depressive disorder; HC, healthy controls.

**Figure 4:**
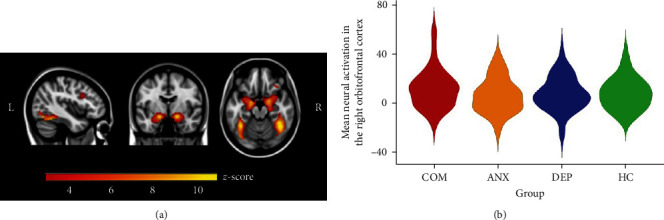
Positive valence system activation. (a) The following contrast was used for task activation: happy emotional faces > scrambled faces. Significant task activation in the bilateral amygdala, hippocampus, fusiform cortex, bilateral frontal gyrus, and right orbitofrontal cortex. Threshold *z* = 3.1 for activation clusters (*x* = −42, *y* = −4, *z* = −18), overlaid on Montreal Neurological Institute 152 2 mm template. (b) The violin plot shows the distribution of the mean neural activation of the significant cluster within the right orbitofrontal cortex in response to positive affect stimuli for each group. COM, comorbid depressive and anxiety disorder; ANX, anxiety disorder; DEP, depressive disorder; HC, healthy controls.

In all participants, the task significantly induced bilateral amygdala, hippocampus, fusiform cortex, frontal gyrus, and right OFC activation (*p* < 0.0001) in reaction to overall negative (angry, sad, and fearful emotional faces) and positive affect (happy emotional faces) stimuli (Figures 3 and 4). These data confirm that the task robustly elicited expected activation across participants. No differences between the four groups were found in mean functional activation across the significant clusters in response to overall negative and positive affect stimuli, in the bilateral amygdala in response to overall negative affect stimuli, or in the right OFC in response to positive affect stimuli (Figures 3 and 4).

**Figure 5:**
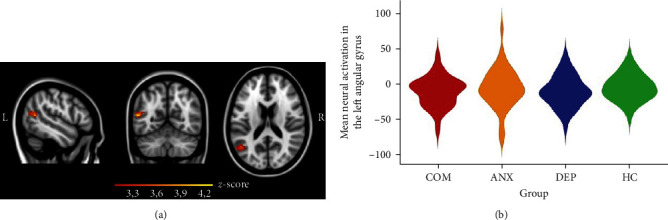
Mean neural activation in the left angular gyrus in response to sad emotion processing. (a) The sagittal, coronal, and axial views are shown (from left to right) of the significant cluster within the angular gyrus (peak coordinates: *x* = −54, *y* = −62, *z* = 12) wherein higher WHODAS scores were coupled with larger activation differences between sad emotional faces > scrambled faces, across depressive, anxiety, comorbid, and healthy control participants. The heatmap corresponds to *z*-values, threshold *z* > 3.1 (FDR corrected) and is overlaid on the Montreal Neurological Institute 152 2 mm template. (b) The violin plot shows the distribution of the mean neural activation of the significant cluster within the angular gyrus for each group. COM, comorbid depressive and anxiety disorder; ANX, anxiety disorder; DEP, depressive disorder; HC, healthy controls.

**Figure 6:**
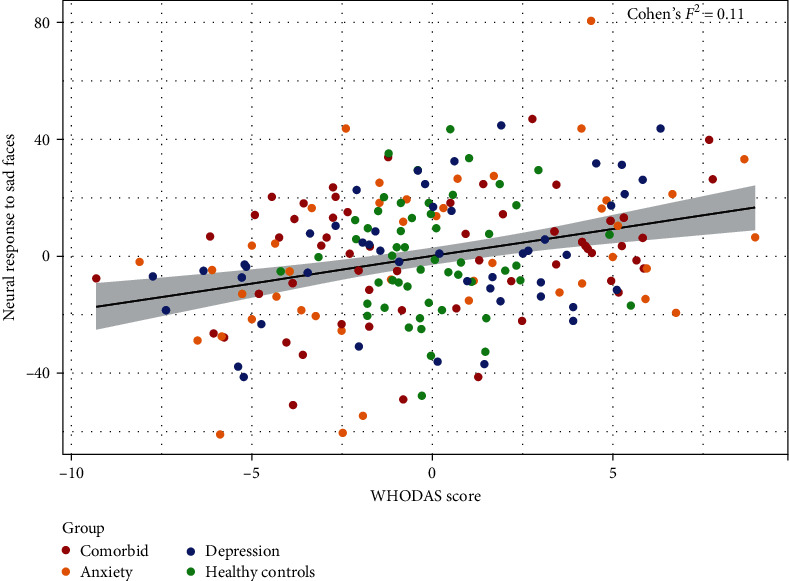
Social dysfunction and sad emotion processing. The partial regression plot provides a quantitative visualization of the association between social dysfunction and sad emotion processing, wherein mean neural responses to sad emotional faces across all voxels of the cluster in the left angular gyrus (peak coordinates: *x* = −54, *y* = −62, *z* = 12) (*y*-axis) are plotted against WHODAS social dysfunction scores (*x*-axis). The values on the *x*- and *y*-axes are residuals after correcting for diagnosis, age, sex, years of education, and scan site. Higher scores on the *x*-axis indicate more severe social dysfunction and higher scores on the *y*-axis indicate larger activation differences between sad emotional faces > scrambled faces. The black solid line depicts the slope of the association across the whole sample, with the gray band indicating the 95% confidence interval of the slope.

The assessment of our a-priori hypothesis that sad emotional valence processing may specifically be associated with social dysfunction based on a previous finding [30], confirmed a similar relationship, whereby greater neural activation in the left angular gyrus (*p*=0.0219, FDR-corrected; *x* = −54, *y* = −62, *z* = 12; *Z*_Max_ = 4.25, Cohen's *F*^2^ = 0.11) in response to sad emotional faces was associated with higher WHODAS scores (i.e., more perceived social disability) across depressive, anxiety, comorbid, and healthy control participants (Figures 5 and 6). Post hoc analyses additionally assessed whether a diagnosis by WHODAS interaction effect or medication (antidepressant and/or benzodiazepine) effects could be identified. The analysis revealed no significant interaction or medication effects (*p* > 0.05).

No significant association was found between neural responses to sad emotional faces and LON scores across the participants (*p* > 0.05). Finally, no significant associations were found between the neural responses to overall negative (fear, sad, and anger) or positive affect stimuli and WHODAS or LON scores across the sample (*p* > 0.05).

## 4. Discussion

The present study aimed to explore a common neurobiological correlate for social dysfunction in the neural circuitry of negative and positive valence processing across persons with and without depressive and/or anxiety disorders. We found that greater functional activation within the left angular gyrus in response to sad emotional faces related to more perceived social disability across all participants, independent of diagnosis. The neural processing of overall negative (sad, angry, and fearful) or positive (happy) valence was not related to perceived social disability across all participants. Additionally, no neural correlates of perceived loneliness were observed.

The findings reported here suggest that the potential negative emotional bias linked to perceived social disability may be more specific to increased neural sensitivity to sad emotional faces instead of a more general bias for negative affect. One may speculate that perceived social disability, here indexed with the WHODAS scale, is more related to sadness rather than anger or fear, based on the theme of the questions of the WHODAS scale (e.g., experiencing difficulties in social contexts). Future studies could investigate whether the more social anxious aspects of social dysfunction are more related to neural activation in response to fearful emotions. Nevertheless, our study showed that greater neural activation within the left angular gyrus in response to sad emotional faces was related to more perceived social disability across all participants. Thus, our findings may suggest that sad emotions are disproportionally seen as salient socioaffective stimuli in the more socially dysfunctional individuals. Previously, we showed that across schizophrenia, Alzheimer's disease, and healthy controls participants, behavioral aspects of social dysfunction were coupled to greater activation within fronto–parieto–limbic regions (e.g., amygdala, insula, ACC, and IPL) in response to sad emotional faces, irrespective of diagnosis [30]. These results partly overlap with the current findings as the bilateral IPL, which includes the angular gyrus, was one of the most significantly associated brain regions in the previous study [30]. Abnormalities in the neural circuitry of the IPL and angular gyrus have been implicated in several mental disorders, including depressive and anxiety disorders, as well as in anhedonia [54–58]. This might indicate that aspects of anhedonia and social dysfunction share a similar neural circuitry. Efforts that aim to reduce levels of anhedonia in mental disorders include treatments with kappa-opioid receptors antagonists, such as buprenorphine [59]. Interestingly, besides reducing levels of anhedonia, buprenorphine has also potential positive effects on social functioning, as it has been shown to reduce the response to social stress, perceived social rejection, and negative emotional stimuli [59–62].

The angular gyrus is a key region of the default mode network (DMN) and has been implicated in many processes, including theory of mind and cognitive reappraisal [63–65]. These processes and the DMN have been implicated in social dysfunction in diverse neuropsychiatric disorders and healthy controls [6, 66–70]. While social cognition has been linked to activation in the bilateral angular gyrus, the left angular gyrus is particularly implicated in episodic memory [71, 72]. This might suggest that sad emotional faces provoke greater neural responses in the left angular gyrus in the more socially dysfunctional individuals, possibly due to a heightened recall of negative emotional memories, although this remains to be investigated. Future research should deepen our understanding of the relationship between altered neural activity of the angular gyrus in response to sad emotional stimuli and social dysfunction by taking aspects of the theory of mind and cognitive reappraisal into account. Interventions may for instance target the cognitive reappraisal process of socioemotional stimuli using transcranial magnetic stimulation (TMS) applied to the angular gyrus, which might indirectly lead to better social functioning outcomes by potentially downregulating the response to negative emotions, although this remains to be investigated [65, 71, 73]. Nevertheless, there is ample evidence in current TMS literature that the angular gyrus plays a causal role in cognition, including episodic memory, information retrieval, and attention [64, 71]. Furthermore, DMN modulation by psychedelics, for instance, psilocybin, also reduces the response to negative affective stimuli, suggesting potential positive effects for social functioning outcomes by targeting 5-HT1 and 5-HT2 receptors [74, 75].

We did not observe an association between neural activity in response to positive valence and social dysfunction. This is not in line with our previous work that showed an association between behavioral aspects of social dysfunction (i.e., social withdrawal and diminished interpersonal functioning) and reduced neural activity in response to happy emotional faces across schizophrenia, Alzheimer's disease, and healthy control participants [30]. A potential explanation is that although the instruments to assess social dysfunction in this previous study and the current study show overlap (Pearson's *r* = 0.662, in a previous study [38]), they capture partly different aspects of social dysfunction (i.e., behavioral aspects of social dysfunction versus perceived social disability). Within the RDoC framework, the positive valence system currently covers systems that are involved in the response to positive motivational situations, such as reward seeking [21]. Therefore, diminished neural responses to happy emotional faces might be more associated with behavioral aspects of social dysfunction that reflect diminished reward seeking, such as social withdrawal, compared to perceived social disability assessed in the current study. Additionally, we found no association between perceived loneliness and neural processing of negative or positive valence across the participants. Previous findings on the relationship between perceived loneliness and (socio-)affective processing have been inconsistent and have not been found in a transdiagnostic manner [30, 66, 70, 76, 77]. Some evidence suggests that the neural circuitry associated with perceived loneliness and a more objective aspect of loneliness, social isolation, might be different [78]. Whether objective social isolation, for instance, assessed via passive smartphone monitoring, is linked to cross-disorder socioaffective neural processing remains to be investigated [39, 79]. The PRISM2 (Psychiatric Ratings using Intermediate Stratified Markers 2) project is aiming to examine this in patients with major depressive disorder, schizophrenia, and Alzheimer's disease in the near future.

The current study focused on the shared neurobiological correlates of social dysfunction in depressive and anxiety disorders, thereby gaining new insights into social dysfunction across these disorders. Previously, it was shown that social dysfunction represents a transdiagnostic domain across these disorders [11]. By showing that this domain has a common neurobiological correlate in depressive and anxiety disorders, we may provide new directions for future personalized treatment irrespective of psychiatric diagnoses. Moreover, our results diverge from previous disorder-specific findings that compared brain activation in response to negative and positive valence between depressive and anxiety disorder patients and healthy controls, further indicating that our findings are distinct and independent of these psychiatric classifications [42]. Nevertheless, the current study had some limitations that need to be acknowledged. First, the cross-sectional design limits our ability to establish the directionality of the relationship between social dysfunction and neural processing of sad emotional faces. Future research that uses TMS or psychotropic medication to normalize the functional and connectional integrity of socioaffective brain systems may increase our understanding of the directionality of the observed association. Second, while the instruments to assess social dysfunction have shown to be valid and reliable across different populations, they are vast simplifications of a complex domain [36, 40, 41]. For instance, as in prior studies, perceived social disability was indexed with five items of the WHODAS scale, which does not capture the multifaceted nature of social dysfunction [38, 39]. Clearly, additional research with more extensive assessments of social dysfunction (e.g., behavioral, affective, and more objective aspects) is needed. For instance, perceived social disability could potentially be measured in more depth and longitudinally using ecological momentary assessment [80]. Additionally, remote monitoring smartphone apps, such as the BeHapp application, may provide more objective measurements of social dysfunction in the future by passively monitoring social exploration and communication [39, 79]. Lastly, due to the implicit nature of the current emotional faces fMRI task, there was no explicit condition to assess whether the emotional content was processed. We opted for an implicit task, because subconscious emotion processing often occurs in daily life, and the implicit emotional faces task may help explore which emotional stimuli are perceived as more salient in this context [81]. Furthermore, since implicit emotional faces tasks (e.g., gender discrimination) are often simpler to perform than explicit tasks that involve emotion categorization, the results can be more easily explored in other neuropsychiatric disorders [82]. Finally, the use of an implicit emotional faces task aligns with our prior work on social dysfunction allowing meaningful comparisons to be made [30]. Future studies could also use an explicit emotional faces task to examine whether negatively biased facial emotion recognition relates to increased neural activation (e.g., in the angular gyrus) in the more socially dysfunctional individuals.

## 5. Conclusions

To conclude, the current study shows that social dysfunction across persons with and without depressive and/or anxiety disorders converges specifically on sad emotion processing of the left angular gyrus. This potential common neurobiological correlate for social (dys)function needs be further validated, for instance, through multimodal (structural, functional, and connectional) examination of the angular gyrus, to achieve a more precise understanding of its contribution to social dysfunction.

## Figures and Tables

**Table 1:** Sample characteristics of each group.

	DEP (*N* = 46)	ANX (*N* = 45)	COM (*N* = 57)	HC (*N* = 52)	Pairwise differences
Demographics					
Age (years), median (Q1–Q3)	35.0 (29.3–44.8)	37.0 (29.0–42.0)	38.0 (26.0–49.0)	40.0 (31.8–47.5)	NS
Sex (female [%])	69.6%	75.6%	66.7%	65.4%	NS
Education (years), median (Q1–Q3)	12.0 (11.0–15.0)	12.0 (11.0–15.0)	11.0 (10.0–12.0)	15.0 (12.0–15.0)	HC > all other groups; ANX > COM
Antidepressant users (%)	23.9%	35.6%	40.3%	0%	—
Benzodiazepine users (%)	4.3%	13.3%	3.5%	0%	—
Number of participants per scan site					
Amsterdam UMC	9	9	9	15	—
UMC Leiden	16	13	28	27	—
UMC Groningen	21	23	20	10	—
Severity disorder					
Depression severity (IDS), median (Q1–Q3)	28.5 (23.0–33.0)	20.5 (11.0–31.0)⁣^*∗*^	32.0 (27.0–39.0)	5.0 (2.0–8.0)	COM > ANX and DEP; all other groups > HC
Anxiety severity (BAI), median (Q1–Q3)	9.5 (5.3–15.0)	14.0 (6.0−19.0)	17.0 (12.0−23.0)	1.0 (0.0–2.0)	COM > DEP; all other groups > HC
Social dysfunction scores					
WHO Disability Assessment Schedule 2.0 (WHODAS), median (Q1–Q3)	13.0 (10.0–15.0)	10.0 (7.0–15.0)	13.0 (10.0–18.0)	5.0 (5.0–6.0)	COM > ANX; all other groups > HC
De Jong-Gierveld loneliness scale, median (Q1–Q3)	5.0 (3.0–8.0)	3.0 (1.0–7.0)	8.0 (4.0–10.0)	1.0 (0.0–2.0)	COM > ANX; all other groups > HC

*Note:* Median and Q1–Q3 are displayed for continuous variables. As assumptions were violated for all continuous variables, a Kruskal–Wallis test with the Dunn's test as a post hoc test was performed. *χ*^2^ tests were performed for categorical variables. A two-tailed significance level of *p* < 0.05 was considered statistically significant.

Abbreviations: ANX, anxiety disorder; BAI, Beck Anxiety Inventory; COM, comorbid depressive and anxiety disorder; DEP, depressive disorder; HC, healthy controls; IDS, Inventory of Depressive Symptomatology; NS, not significant.

⁣^*∗*^*N* = 44; the IDS score of *N* = 1 patient in the anxiety group was missing.

## Data Availability

The data used to support the findings of this study are available upon reasonable request from NESDA, Amsterdam: nesda@amsterdamumc.nl. Information on how to request the study data, including the data-sharing policy, can be found at https://www.nesda.nl/nesda-english/.
